# Breakfast Frequency and Sleep Quality in College Students: The Multiple Mediating Effects of Sleep Chronotypes and Depressive Symptoms

**DOI:** 10.3390/nu15122678

**Published:** 2023-06-08

**Authors:** Xiaobing Xian, Chunyuan Wang, Rong Yu, Mengliang Ye

**Affiliations:** 1 School of Public Health, Chongqing Medical University, Chongqing 400016, China; 2020222012@stu.cqmu.edu.cn (X.X.); 2020221937@stu.cqmu.edu.cn (C.W.); 2 School of Traditional Chinese Medicine, Chongqing Medical University, Chongqing 400016, China; 2022221021@stu.cqmu.edu.cn

**Keywords:** breakfast frequency, sleep chronotypes, depressive symptoms, sleep quality

## Abstract

Sleep disorders, which are prominent problems among college students, may be associated with skipping breakfast. Therefore, we aimed to explore the role of sleep chronotypes and depressive symptoms as mediators in the relationship between breakfast frequency and sleep quality. A cross-sectional survey enrolling random samples of 712 college students was conducted by the Questionnaire Star online platform. Statistical description and correlation analysis were performed by SPSS 25.0, and a chain mediation test was performed by model 6 in PROCESS 3.5. The result of the article demonstrated that breakfast frequency can affect sleep quality through two mediating pathways: ① sleep chronotypes, with a mediating effect of 32%; and ② depressive symptoms, with a mediating effect of 52.4%. However, the chain mediating effects of sleep chronotypes and depressive symptoms was not significant, and neither was the direct effect of breakfast frequency on sleep quality. Breakfast frequency can indirectly affect sleep quality by adjusting sleep chronotypes and depressive symptoms. Regular breakfast can increase morning and intermediate sleep chronotypes, reduce depressive symptoms, and thus improve sleep quality.

## 1. Introduction

During the COVID-19 pandemic, in order to make up for the shortage of medical and health resources and better maintain public health and social order, many countries adopted quarantine, including studying online for college students. At home, without the supervision of the school and peer influence, college students are more likely to be influenced by self-control, such as sleeping early or late, eating breakfast or not, and so on. Furthermore, we found that during online classes at home, students’ diet and lifestyle habits changed dramatically, including the longer use of electronic devices, lesser breakfast frequency, and longer sleep duration [1]. At the same time, online classes at home also have different degrees of impact on the mental health and sleep chronotype (SC) of college students [2]. Relevant studies have shown that depressive symptoms (DS) and anxiety are often associated with lower sleep quality (SQ) [3], and different sleep chronotypes (SC) also predict different SQ [4]. Regular nutritious breakfast is conducive to reducing the occurrence of DS, regulating SC, and improving SQ [5,6,7,8]. Although the relationship between breakfast frequency (BF) and SQ has been demonstrated by other scholars, the mechanism for the effect of BF on SQ remains unclear. Based on the above, we hypothesize that SC and DS are the two influential mediators.

### 1.1. Breakfast Frequency and Sleep Quality

Numerous studies have linked breakfast to nutritional intake, weight control, cardio metabolic risk factors, and cognitive performance [9]. Furthermore, one review suggests that skipping breakfast may reduce free-living physical activity and endurance exercise performance throughout the day [10]. The importance of breakfast is self-evident; a relevant study also showed that eating habits and meal timing were significantly associated with SQ, as eating at inappropriate times or skipping meals is responsible for disturbances in the peripheral circadian clock and metabolism. At the same time, people with poor SQ or insufficient sleep time are more likely to show irregular eating patterns such as skipping breakfast [5,11]. The study conducted by Zhang Y et al. also contends that good sleep is related to regular breakfast [12]. However, during the coronavirus pandemic, the frequency of college students skipping breakfast increased [13,14]. During online classes, many college students often chose to skip breakfast in exchange for more sleep. Even if we are not in such a special period, it is still common for college students to skip breakfast. Sleep health is a multidimensional construct that includes objective quality (e.g., sleep efficiency), subjective quality, and variability, all of which predict breakfast consumption [11]. Good sleep health is associated with regular BF, so improving SQ can often start with getting into the habit of eating breakfast regularly.

### 1.2. Sleep Chronotypes and Sleep Quality

The circadian system regulates many aspects of eating behavior, including hunger rhythms, food intake timing, and so on. SC is a self-assessed description of circadian preferences that reflects the organization of the circadian system. Researchers generally distinguish three main SCs: morning (“larks”), intermediate, and evening (“owls”) [15]. SCs and SQ have been shown to play significant roles in people’s physical and mental health. College students’ SC can often predict SQ. Morning-type students and intermediate-type students had a lower risk of poor SQ compared to evening types [7,16]. Evening-type students go to bed later with more bedtime procrastination and experience lower SQ [4]. From the other side, Romo-Nava et al. show that in individuals with bipolar disorder, the evening chronotype is associated with unhealthy dietary patterns such as breakfast skipping [17]. Generally speaking, people who skip breakfast tend to be more inclined to be evening types [8]. Therefore, it is believed that SQ among evening-type students may be improved by shifting to keeping early hours [7].

### 1.3. Mediating Role of Depressive Symptoms

Depression, and its associated mood disorders, is a global health problem and was one of the most common psychiatric disorders during the COVID-19 pandemic [18]. For adolescents, depression is also a major risk factor for suicide. Therefore, we have to admit that the prevention and control of DS is necessary. During the pandemic, college students may have been particularly at an increased risk of mental illness due to university closures, academic disruptions, and social restrictions, indirectly affecting the quality of sleep of college students [19]. The decrease in SQ of college students further leads to the increase in negative emotions, such as DS and anxiety. Research has shown a potential relationship between skipping breakfast and mood problems in adolescents, which leads to lower cognitive and emotional engagement [15]. Compared with eating breakfast, skipping breakfast is strongly associated with anxiety and DS [20,21]. Xu, Y. et al. also demonstrated that better and longer duration of sleep and the regular eating of breakfast can help reduce the occurrence of DS and help in the development of a healthy lifestyle [6]. Therefore, it is reasonable to speculate that DS can be used as a mediating factor in BF affecting SQ.

Based on the above studies, we can speculate that there is a relationship between BF, DS, SC, and SQ. However, the impact mechanism of BF on SQ is not clear, so we aimed to investigate the relationship between the above four and to explore the multiple mediating roles of DS and SC between BF and SQ. We investigated the situation of college students in Chongqing Medical University’s online classes and fully explored the mechanism of BF affecting SQ, aiming to provide some guidance for college students to develop a healthy lifestyle and promote public health. Therefore, we proposed the following four hypotheses:

**Hypothesis 1 (H1).**  *BF has an impact on SQ.*

**Hypothesis 2 (H2).**  *BF affects SQ through the separate mediating effect of SC.*

**Hypothesis 3 (H3).**  *BF affects SQ through the separate mediating effect of DS.*

**Hypothesis 4 (H4).**  *BF affects SQ through the chain mediating effect of SC and DS.*

## 2. Materials and Methods

### 2.1. Participants and Procedure

This study used a cross-sectional survey method. From 19 September 2022 to 27 September 2022, 712 college students from Chongqing Medical University were recruited to participate in an online questionnaire survey using the convenient sampling method. The questionnaire included basic demographic variables, social support scale (PSSS), depression–anxiety–stress scale (DASS-21), the Pittsburgh Sleep Quality Index (PSQI) scale, and morning and evening questionnaire (MEQ-5). Before the survey, professional investigators introduced the survey content and purpose to obtain all participants’ informed consent. Questionnaire filling, data collection, and summary were all performed on the Questionnaire Star online platform; the inclusion criteria were sophomores and above, and having online class experience, and the exclusion criteria were no history of major diseases, chronic diseases, or trauma, and incomplete questionnaires. After removing missing and invalid data, the number of remaining samples was 660.

### 2.2. Measurement

#### 2.2.1. Breakfast Frequency

We measured this through the question “How many days have you had breakfast in the past week?” to measure how often participants ate breakfast. Results ranged from 0 to 7, and the higher the value, the higher the frequency of breakfast.

#### 2.2.2. Sleep Chronotypes

The morning and evening questionnaire (MEQ) is currently the internationally accepted classification tool for the natural trend of sleep–wake circadian rhythm, which belongs to the category of self-assessment of sleep behavior. We used the morning and evening scale (MEQ-5) to assess SC, and a simplified 19-item version of the classic 5-item MEQ to assess chronotypes, where the scores of the 5 items are added together, with higher scores representing earlier chronotypes. This questionnaire has validity and reliability to a certain extent, and is widely used in the assessment of sleep [22]. Furthermore, the Cronbach coefficient was 0.744.

#### 2.2.3. Depressive Symptoms

DS was measured using the depression component of the depression–anxiety–stress scale (DASS-21). Brown et al. (1997) demonstrated that the DASS scale could be reliably categorized into depression (DASS-D), anxiety (DASS-A), and stress (DASS-S). The depression subscale assessed lack of incentive, low self-esteem, and dysphoria. Compared to the traditional DASS-42 scale, DASS-21 has a greater differential validity [23]. The Cronbach coefficient for the depression subscale in this study was 0.847, and more than 0.8 means a study with higher reliability.

#### 2.2.4. Sleep Quality

The Pittsburgh Sleep Quality Index (PSQI) is a retrospective self-assessment questionnaire, mainly used to measure SQ in the previous month. The scale consists of 18 self-assessment items and 7 components. Component scores range from 0 (no difficulty) to 3 (severe difficulty), and each part is added together to produce a global score ranging from 0 to 21 [24]. A global score higher than 5 is considered to be an indicator of relevant sleep disturbances in at least two components, or of moderate difficulties in more than three components, and a higher score indicates worse SQ. The Cronbach coefficient in this study was 0.837.

#### 2.2.5. Basic Demographic Variables

To minimize the bias in the results, we performed measures of basic demographic characteristics, including sex, age, grade, height, weight (calculated body mass index (BMI)), monthly family income, and home address.

### 2.3. Statistical Analysis

For frequency and percentage representation of categorical variables (*n* (%)), continuous variables used mean and standard deviation (M ± SD). The normality test and homogeneity test of variance were conducted for the scores of BF, SC, DS, and SQ under different basic demographic characteristics, and the t test of two independent samples or homogeneity-of-variance were further conducted to determine whether there were differences in the mean values of BF, SC, DS, and SQ among different subgroups when all were satisfied. This study used SPSS 25.0 to process data and perform correlation analyses on BF, SC, DS, and SQ. The Harman univariate test was used for common bias test. Furthermore, the multiple mediation effect model was tested using model 6 in the PROCESS program developed by Hayes, and the significance of the mediation effect was tested by sampling 2000 times by bias-corrected percentile bootstrap. The significance level is 0.05.

## 3. Results

### 3.1. Basic Demographic Differences

As shown in Table 1, BF scores varied significantly across BMI (F = 3.994, *p* = 0.019). SC scores varied by gender, age, monthly family income, and home address (t = −3.832, *p* < 0.000; t = −2.723, *p* = 0.007; t = 2.621, *p* = 0.009; t = 2.287, *p* = 0.023, respectively). Finally, the mean of DS and SQ scores also differed significantly by gender (t = −2.244, *p* = 0.025; t = 2.147, *p* = 0.032).

### 3.2. Common Deviation Test

The specific method of testing is to employ exploratory factor analysis on all topics to see if the explanatory amount of the precipitated first common factor is below the critical value of 40.0%. This study precipitated a total of six common factors with feature values greater than 1. Among them, the amount of explanation of the first common factor is 22.80%, which is below the critical value, which means that the data are acceptable without significant common method deviation.

### 3.3. Pearson Correlation Analysis

The results of Pearson correlation analysis showed that BF and SC were positively correlated (r = 0.194, *p* < 0.001), while BF and DS as well as BF and SQ were negatively correlated (r = −0.112, *p* < 0.01; r = −0.083, *p* < 0.05, respectively). SC and SQ were negatively correlated (r = −0.165, *p* < 0.01), and SC and DS were not correlated (r = −0.027). DS and SQ were positively correlated (r = 0.376, *p* < 0.01). The detailed results are shown in Table 2.

### 3.4. Regression Analysis and Chain Mediated Effects Test

Based on the correlation of BF, SC, DS, and SQ, we used multiple mediation analysis to explore the mediating role of SC and DS in the college student population. With BF as the independent variable (X), SC and DS as mediating variables (M), and SQ as the dependent variable (Y), a chain mediation model was constructed. The analysis results in Table 3 show that BF and SC were positively correlated (a1 = 0.184, *p* < 0.001), while BF and DS were negatively correlated (a2 = −0.111, *p* < 0.01). There was an inverse correlation between SC and SQ (B1 = −0.140, *p* < 0.001), and DS and SQ were positively correlated (B2 = 0.380, *p* < 0.001). BF was not significantly associated with SQ, and SC was not significantly associated with DS. Regression models incorporating BF, SC and DS as independent variables explained a total of 17.5% of the variance in sleep quality (R2 = 0.175).

**Figure 1:**
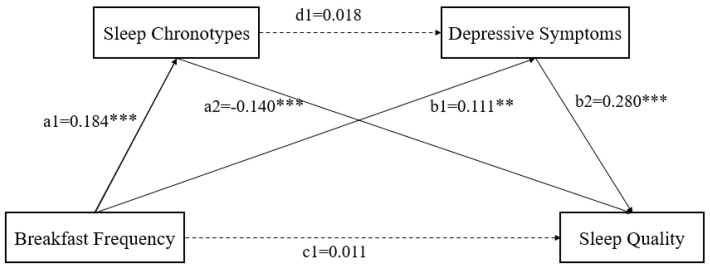
A chain mediation model through the association between breakfast frequency, sleep chronotypes, depressive symptoms, and sleep quality. Path coefficients are shown. Note: ** *p* < 0.01, and *** *p* < 0.001.

The mediation path model is shown in Figure 1. The path coefficient shows that all relationships in the model are significantly positive or negative. BF indirectly affects SQ through two mediations: SC and DS.

To test whether the mediation effects of SC and DS was significant, we designed an autonomous estimation procedure for the samples. The effect is the most when the pathway coefficient of the 95%CI does not include 0. Table 4 lists the total, direct, and indirect effects. As shown in Table 4, when mediating factors were incorporated into the model, the direct effect of BF on SQ was not significant, while BF was found to indirectly affect SQ through two significant mediating pathways: (1) SC (B = 0.012, 95%CI = −0.016, −0.012), accounting for 32.0% of the total effect, and (2) DS (B = 0.020, 95%CI = −0.095, −0.017), accounting for 52.4% of the total effect. However, the chain mediating effect of SC and DS was not significant.

## 4. Discussion

Although there are many studies on the factors related to SQ and BF, the mechanism of how BF affects SQ has not been fully elucidated. In this study, we established a multiple intermediary model to show how the relationship between BF and SQ transmitted through DS and SC. We first described the demographic characteristics and found that there were some differences in BF, SC, DS, and SQ among different populations. Furthermore, we established three main pathways: DS as a separate mediator, SC as a separate mediator, and DS and SC as multiple mediators. The results show that the direct effect of BF on SQ is not significant, but the indirect effects tellingly show. The separate mediation effect of DS and SC is obvious, but the multiple mediation effects of DS and SC are not significant.

### 4.1. Differences under Different Subgroups of Different Basic Demographic Characteristics

We found body mass index (BMI) varies from different BFs. According to the results of other studies, BF may be related to eating habits, and people with an unhealthy diet have higher BMIs than those with healthy eating habits [25], which is consistent with our results. SC varies by age and monthly family income. Related studies have shown that the older people are, the more likely they are to be evening types, the later the rising time and bedtime they have [26]. In addition, the low-income population may be more inclined to the evening chronotype [27], but we cannot investigate the income level of all participants in this study because they are college students and receive money from their parents; therefore, a further study is needed. In addition, DS scores were significantly different by gender. Further, the gender gap in adolescent DS increases over time, with females experiencing a higher rate of DS [28]. Finally, SQ scores varied significantly from gender and grade, and the gender differences are more significant. Female students have lower SQ scores than male students. On weekdays, freshmen woke up earlier and slept shorter than other students [29]. In summary, the gender and age factors were the most significant differences in our study, so we analyzed them as control variables.

### 4.2. Effect of Breakfast Frequency on Sleep Quality

In previous studies, there was a correlation between BF and SQ. This is consistent with our assumptions. Sleep time is so crucial to breakfast intake that people with poor SQ will be more inclined to irregular eating habits [11,30,31,32]. At the same time, skipping breakfast is also related to a variety of factors, especially lack of sleep [32]. However, previous studies tested BF and SQ as part of multiple variables, and only a few scholars focused on the influencing mechanisms of BF on SQ. With the development of social life, people are increasingly advocating a healthy lifestyle, including regular diet and high-quality sleep. In this study, BF did not directly affect SQ, and we suspect the following reasons: firstly, our sample size was not large enough and the non-probabilistic convenience sampling method may be biased. Secondly, the survey was conducted during the COVID-19 lockdown, the SQ of college students was affected by a variety of factors, and the impact of BF was not prominent in comparison. Finally, the methodology used in some of the studies was a cohort study, which had less chance of bias in comparison. However, we found that BF can indirectly affect SQ from other pathways, and the indirect effect was 85.4%. We will discuss the mechanism by which BF affects SQ from the perspective of DS and SC.

### 4.3. Mediator Role of the Sleep Chronotypes

The study found that SC also played a mediating role in the relationship between BF and SQ, with a mediating effect of 32.0%. This is in agreement with previous studies: people who skip breakfast tend to sleep late and tend to sleep later in a lower sleep quality [33,34,35]. In addition, people who sleep later or sleep poorly are more likely to change their eating habits, such as missing or not being in time for meals, which also indicates a higher frequency of skipping breakfast. It is obvious that the evening type is more likely to skip breakfast, which is consistent with previous research [36,37,38,39,40]. Different SCs represent different active hours: morning and intermediate types tend to be active in the morning or during the daytime, while evening types are active late at night. However, too much nocturnal activity time for college students who are enrolled in morning classes can lead to their later sleep onset and shorter sleep time, so SQ is inevitably decreased. For college students, actively increasing the amount of time spent awake in the morning (such as increasing the frequency of breakfast) may benefit the adjustment of their SC—the frequency of the morning and intermediate types is increased, which is conducive to improving their SQ.

### 4.4. Mediating Role of DS

This study found that DS played a mediating role between BF and SQ, with a mediating effect of 52.4%, which is consistent with previous findings. People who skip breakfast are more likely to show higher symptoms of stress and depression than those who eat breakfast, and there is a significant positive correlation between skipping breakfast and the odds ratio of depression, stress, and psychological distress [41,42,43,44,45]. At the same time, there is also a mutual effect between mental health problems such as DS and SQ; DS will reduce SQ, and reduced SQ will also lead to mental health problems such as DS [46,47]. At present, DS among college students is still relatively common. Based on the above conclusions, we believe that DS can act as a mediator of BF in regulating SQ. Therefore, we suggest that college students should develop a habit of eating breakfast, obtaining high SQ, and maintaining a healthy lifestyle to protect themselves against DS.

### 4.5. Chain Multiple Mediation Model of Sleep Chronotypes with Depressive Symptoms

Similarly, we also propose a chain multiple mediation model of SC with DS, and there is no significant chain between SC and DS from the model regression and correlation analysis. However, previous studies have proved the relationship of SC and DS and revealed the interaction of evening types and DS; evening types are more prone to be depressive and suicidal. Meanwhile, people experiencing DS and suicidality also show greater preference to being evening types, but the mechanism is still unclear [48,49,50]. Obviously, this is inconsistent with our results, because the method we used was a questionnaire survey, which is less precise and has a higher error compared to the self-assessment and diagnostic interview methods. Additionally, compared with previous studies, our sample size was smaller and the significance level was lower. Therefore, that is what we are going to improve in future research.

In summary, regular diet and high-quality sleep are positively related to the physical and mental health as well as the healthy lifestyle formation of college students. We discussed the two influence pathways of BF on SQ via SC and DS, and we revealed that regular breakfast is a feasible and effective way for college students to keep fit.

## 5. Conclusions

This study provides empirical evidence that BF has an indirect impact on SQ in the regulation of SC and DS, and the results of this study suggest that college students should develop a habit of regular breakfast, which is conducive to increasing morning and intermediate SC, reducing DS, improving SQ, and thus staying healthy rather than skipping breakfast to be vexed by sleep disorders.

## 6. Limitations

Our hypotheses are still partially unproven, and our study has some limitations. Firstly, the descriptive studies we cited were difficult to determine the causal and temporal relationship between BF and SQ from, and the sample survey was prone to selection bias and information bias. Therefore, cohort studies can be conducted on existing conclusions for further discussion on the causal and temporal relationship among the variables. Secondly, due to the COVID-19 quarantine, the generalizability of this study may be influenced by the uncertain changes on psychology and obtaining of food of college students, especially those living alone. In addition, during the COVID-19 period, in order to fulfill limited duties, the social activities of college students were restricted, which may have led to an increase in evening chronotype and result in some bias in the results. Thirdly, the main purpose of our study was to investigate the influencing mechanisms of BF on SQ, while the lack of covariates on sleep quality may lead to bias in the results. Finally, the experimental results may widely vary in college students from different regions and skin colors, while all participants in this study were only from Chongqing Medical University because of the COVID-19 lockdown. Hence, generalization of the study findings requires further validation using a larger, more representative sample study.

## Figures and Tables

**Table 1:** Differences in breakfast frequency, sleep chronotypes, depressive symptoms, and sleep quality under different subgroups of basic demographic characteristics.

Variable	*n* (%)	Breakfast Frequency	t/F (*p*)	Sleep Chronotypes	t/F (*p*)	Depressive Symptoms	t/F (*p*)	Sleep Quality	t/F (*p*)
Gender Male Female	187(28.3) 473(71.7)	4.68 ± 2.21 4.41 ± 2.29	−1.414 (0.158)	12.18 ± 3.18 14.17 ± 2.98	−3.832 (<0.000)	7.78 ± 7.5 6.49 ± 6.25	−2.244 (0.025)	4.24 ± 3.26 4.77 ± 2.72	2.147 (0.032)
Grade Sophomore year Junior year Senior year Above	287(43.5) 273(41.4) 56(8.5) 44(6.7)	4.39 ± 2.23 4.47 ± 2.30 4.64 ± 2.28 5.16 ± 2.15	1.047 (0.382)	14.40 ± 3.00 14.31 ± 3.09 14.75 ± 3.11 15.55 ± 3.23	1.539 (0.189)	6.79 ± 6.34 7.01 ± 7.06 7.29 ± 6.36 5.79 ± 6.29	0.294 (0.813)	4.60 ± 2.82 4.52 ± 2.85 4.80 ± 3.26 4.87 ± 2.96	2.147 (0.032)
BMI <18.5 18.5~23.9 >23.9	140(21.2) 444(67.6) 76(11.2)	4.36 ± 2.28 4.84 ± 2.14 6.25 ± 1.50	3.994 (0.019)	14.35 ± 2.96 14.78 ± 3.34 15.00 ± 4.40	1.298 (0.274)	6.75 ± 6.46 7.07 ± 7.11 11.50 ± 11.12	1.124 (0.326)	4.73 ± 2.84 4.22 ± 2.95 6.50 ± 4.80	1.240 (0.293)
Age ≤20 >20	447(67.7) 213(32.3)	4.44 ± 2.30 4.58 ± 2.20	−0.750 (0.454)	14.23 ± 2.98 14.92 ± 3.20	−2.723 (0.007)	6.95 ± 6.70 6.66 ± 6.55	0.526 (0.599)	4.56 ± 2.83 4.74 ± 3.01	2.808 (0.061)
Monthly family income ≤5000 >5000	368(55.8) 292(44.2)	4.39 ± 2.22 4.60 ± 2.31	−1.176 (0.230)	14.73 ± 3.04 14.11 ± 3.08	2.621 (0.009)	7.32 ± 6.86 6.27 ± 6.34		4.77 ± 2.87 4.42 ± 2.91	−0.730 (0.466)
Home address Township City	281(42.6) 379(57.4)	4.40 ± 2.25 4.55 ± 2.28	−0.892 (0.373)	14.77 ± 3.07 14.22 ± 3.05	2.287 (0.023)	7.27 ± 6.76 6.55 ± 6.56	1.373 (0.170)	4.81 ± 2.95 4.48 ± 2.84	1.454 (0.147)

**Table 2:** Pearson correlation analysis of breakfast frequency, sleep chronotypes, depressive symptoms, and sleep quality.

Variable	Breakfast Frequency	Sleep Chronotypes	Depressive Symptoms	Sleep Quality
Breakfast frequency	1			
Sleep chronotypes	0.194 ***	1		
Depressive symptoms	−0.112 **	−0.027	1	
Sleep quality	−0.083 *	−0.165 **	0.376 **	1

Note: * *p* < 0.05, ** *p* < 0.01, and *** *p* < 0.001.

**Table 3:** Regression analysis of breakfast frequency, sleep chronotypes, depressive symptoms, and sleep quality.

Regression Model		Model Fit Index			Significance of Regression Coefficients	
Outcome Variables	Predictive Variables	R	R2	F	β	t
Sleep Chronotypes	Breakfast frequency	0.239	0.057	13.200	0.184	11.646 ***
Depressive symptoms	Sleep chronotypes	0.149	0.022	3.731	−0.018	−0.456
	Breakfast frequency				−0.111	−2.803 **
Sleep quality	Sleep chronotypes	0.418	0.175	27.755	−0.140	−3.818 ***
	Breakfast frequency				−0.011	−0.310
	Depressive symptoms				0.380	10.586 ***

Note: ** *p* < 0.01, and *** *p* < 0.001.

**Table 4:** Significance test for mediating effects of breakfast frequency, sleep chronotypes, depressive symptoms, and sleep quality.

	Effect	BootSE	BootLLCI	BootULCI	Percentage of Total Effect
Total effect	−0.103	0.050	−0.200	−0.005	100%
Direct effect	−0.015	0.047	0.756	−0.106	__
Total indirect effects	−0.088	0.024	−0.141	−0.044	85.4%
Breakfast frequency → sleep chronotypes → sleep quality	−0.033	0.012	−0.061	−0.012	32.0%
Breakfast frequency → depressive symptoms → sleep quality	−0.054	0.020	−0.095	−0.017	52.4%
Breakfast frequency → sleep chronotypes → depressive symptoms → sleep quality	−0.002	0.004	−0.010	0.007	__

## Data Availability

The datasets used and analyzed in this study are available from the corresponding author upon request.
